# Disparities in the change of cervical cancer mortality rate between urban and rural Chiang Mai in the era of universal health care and the Thai national screening program

**DOI:** 10.1186/s12939-021-01515-1

**Published:** 2021-07-29

**Authors:** Patumrat Sripan, Imjai Chitapanarux, Ekkasit Tharavichitkul, Pooriwat Muangwong, Donsuk Pongnikorn, Narate Waisri, Chirapong Hanpragopsuk, Puttachart Maneesai, Panrada Tansiri, Malisa Poungsombat, Varunee Khamsan

**Affiliations:** 1 Research Institute for Health Sciences, Chiang Mai University, Chiang Mai, Thailand; 2 Northern Thai Research Group of Radiation Oncology (NTRG-RO), Faculty of Medicine, Chiang Mai University, Chiang Mai, Thailand; 3 Division of Radiation Oncology, Department of Radiology, Faculty of Medicine, Chiang Mai University, Chiang Mai, Thailand; 4 Chiang Mai Cancer Registry, Faculty of Medicine, Chiang Mai University, Chiang Mai, Thailand; 5 Cancer Registry Unit, Lampang Cancer Hospital, Lampang, Thailand

**Keywords:** Area of residence, Cervical cancer, Disparity, Mortality trend, Screening

## Abstract

**Background:**

The Ministry of Public Health of Thailand established universal health coverage (UHC) in 2002, which also included national-level screening for cervical cancer in 2005. This study examined the changes in mortality of cervical cancer in rural and urban areas in Chiang Mai Province of northern Thailand during the era of UHC and the immediately preceding period.

**Methods:**

Data of cervical cancer patients in Chiang Mai in northern Thailand, who died from 1998 through 2012, were used to calculate the change in age-standardized rates of mortality (ASMR) using a joinpoint regression model and to calculate estimated annual percent changes (APC). The change in mortality rate by age groups along with changes by geographic area of residence were determined.

**Results:**

Among the 1177 patients who died from cervical cancer, 13(1%), 713 (61%) and 451 (38%) were in the young age group (aged < 30), the screening target group (aged 30–59) and the elderly group (aged ≥60), respectively. The mortality rate among women aged 30–59 significantly declined by 3% per year from 2003 through 2012 (*p* < 0.001). By area of residence, the mortality rate in women targeted by the screening program significantly decreased in urban areas but remained stable in more rural areas, APC of − 7.6 (95% CI: − 12.1 to − 2.8) and APC of 3.7 (95% CI: − 2.1 to 9.9), respectively.

**Conclusion:**

The UHC and national cervical cancer screening program in Thai women may have contributed to the reduction of the mortality rate of cervical cancer in the screening target age group. However, this reduction was primarily in urban areas of Chiang Mai, and there was no significant impact on mortality in more rural areas. These results suggest that the reasons for this disparity need to be further explored to equitably increase access to cervical cancer services of the UHC.

**Supplementary Information:**

The online version contains supplementary material available at 10.1186/s12939-021-01515-1.

## Introduction

A key objective of health systems worldwide is equitable access to the health system regardless of ability to pay [1]. In 2002, universal health coverage (UHC) was launched in Thailand and covered approximately 47 million people or 75% of the entire population who were not previous beneficiaries of either the Civil Servant Medical Benefit Scheme (CSMBS) or the Social Security Scheme (SSS) [2]. This program aimed to reduce socioeconomic inequalities in urban and rural health service use in Thailand. Between 2001 and 2005, the UHC substantially reduced Thailand’s uninsured population from 42.5 to 7.0% in urban areas and from 24.9 to 2.7% in rural areas [3].

In 2005, a national screening program for cervical cancer was implemented as part of the UHC package offered to all Thai women aged 30–60 years, involving screenings every 5 years, in order to reduce the burden of cervical cancer [4, 5]. The National Health Security Office (NHSO) established a contract with the Ministry of Public Health to provide both Papanicolaou smear (Pap Smear) and visual inspection with acetic acid (VIA) as eligible benefits covered under the universal health coverage plan, allowing health care providers to choose the approach they considered to be the most appropriate to their setting [5, 6].

In Thailand, cervical cancer was once the top cancer in women until 2002, with an age-standardized incidence rate (ASIR) of 20.7 per 100,000 women and mortality rate (ASMR) of 3.53 per 100,000 women-year. In 2020, the incidence rate dropped to 11.3 women-year but mortality rate was 4.1 per 100,000 women [7, 8]. Following implementation of the Thai national cervical cancer screening program targeting women aged 30–59 years of age, the incidence rates of cervical cancer decreased nationally. There was also a downshifting in the stage distribution of malignant tumors following the launch of this program. This evidence supported that the efforts to reduce the burden of cervical cancer have been successful. These changes have also been seen in women in Chiang Mai, evidence that the screening program was successful outside of Bangkok, with a similar downshifting of the stage distribution of malignant tumors and a reduction in incidence of malignant tumors as more in-situ cases were captured [9]. In terms of survival, the inclusion of cervical cancer screening into the UHC has likely reduced the magnitude and severity of cervical cancer, and improved the survival of patients in the screening target age group [10].

Difference in the mortality rate of cervical cancer between urban and rural areas has been reported both in low-resource and high-income countries. In the United State of America from 1969 to 2007, both white and black women in non-metropolitan areas maintained significantly higher cervical cancer mortality rates compared to their metropolitan counterparts. Among black women, cervical cancer mortality declined at a faster pace in metropolitan compared to non-metropolitan areas [11]. Similar results were found in Mexico, where women living in rural areas had higher cervical cancer mortality risks compared to women with an urban residence [12]. However, the change of these patterns of mortality, which is one major indicator of success has not been reported in the setting of Thailand. Our study is the first population-based study showing this indicator.

The main objective of this study is to examine changes in mortality of cervical cancer between rural and urban areas of Chiang Mai Province of northern Thailand in the era of UHC during the advent of the national screening program for cervical cancer and the immediately preceding period.

## Materials and methods

### Data

Data of patients who died from cervical cancer during the periods immediately preceding and following implementation of UHC were extracted from the Chiang Mai Cancer Registry based on a search for the International Classification of Diseases 10th edition (ICD-10) code for cervical cancer (C53). The first period, prior to advent of UHC, was in 1998–2002, and the second, during the advent of the UHC, was from 2003 to 2012. Individual cancer registration records extracted included date of birth, age at diagnosis, clinical diagnosis, pathological reports, clinical extent of disease before treatment, and initial treatment modality for adults (aged 15–99 years). Mortality data was obtained from the Bureau of Registration Administration, Ministry of Interior, of Thailand. Deaths from cervical cancer occurring at any hospital in Chiang Mai had cause of death confirmed using data from the Chiang Mai Cancer Registry.

### Population

Chiang Mai Province is located approximately 600 km north of Bangkok in northern Thailand, occupying an area of 20,107 km^2^. The Chiang Mai Cancer Registry covers 25 districts and accounts for 15% of the population of northern Thailand as a whole. The population of Chiang Mai Province based on the 2010 census was approximately 1.7 million people, of whom 51% were female. For the calculation of incidence rates, population data was obtained from the Official Statistics Registration Systems, Department of Provincial Administration, where data on population size by age group and area of residence were available. Specifically, the populations of the year at the middle of each 5-year periods (three periods) were used as denominators: 2000 for period I (1998–2002), 2005 for period II (2003–2007) and 2010 (2008–2012) for period III.

Analyses were conducted for all women and categorized by three age groups: women aged < 30 years and thus younger than the targeted screening program population, women 30–59 years old (the target population for screening), and those aged ≥60 years (older than the targeted screening program population) to determine cervical cancer mortality rate trends. Muang District, which houses the main Chiang Mai city metropolitan area, and the districts immediately bordering it including Saraphi, San Sai, San Kamphaeng, Mae Rim, Doi Saket and Hang Dong, were defined as urban based on their short distance from the city center [13].

### Statistical analysis

Age-standardized rates (ASR) for the mortality of cervical cancer were calculated for 5-year intervals of age groups ranging from 15 to 19 to 80–84, and then for 85 years and older. ASRs standardized to the world population proposed by Segi [14], and later modified by Doll [15], were computed for each particular year using the Joinpoint Regression Program, version 4.5.0.1 (Surveillance Research Program National Cancer Institute, 2017), then plotted to visually illustrate the trends using R Program. A Joinpoint regression model was used to estimate the percentage of change (annual percent change or APC) using the log transformation of the response. The statistically significant change points was identified at 0.05 significant level in each trend segment using a Monte Carlo permutation method [16]. The Permutation test was also used to determine the best number of joinpoints.

This study was approved by the Research Ethics Committee of the Faculty of Medicine of Chiang Mai University (Approval number: 449/2017).

## Results

Our study included 1177 patients who died of cervical cancer in Chiang Mai during the period immediately prior to implementation of UHC (1998–2002) and after (2003–2012). The Table 1 shows the characteristics of the patients by periods of death. The median age at diagnosis and age at death were both higher, and less than 15% of area of residence data was missing following advent of UHC. During the era of UHC (2003–2012), among women of screening program age, area of residence was not associated with both age at diagnosis and age at death as well as the period of diagnosis (Table 2).

**Table 1:** Characteristics of patients in Chiang Mai, Thailand, who died of cervical cancer before (1998–2002) and after (2003–2012) the implementation of universal health coverage

	Before UHC (1998–2002) *N* = 355	After UHC (2003–2012) *N* = 822	*p*-value	Total *N* = 1177
**Characteristics**				
Median (IQR) age at diagnosis (year)	52 (42–61)	56 (48–68)	< 0.001^a^	55 (46–66)
Age group, n(%)			< 0.001^b^	
15–29	8 (2)	5 (1)		13(1)
30–59	239 (67)	474 (58)		713 (61)
≥ 60	108 (30)	343 (42)		451 (38)
Median (IQR) age at death (year)	54 (45–65)	60 (51–71)	< 0.001^a^	58 (49–69)
Age group, n(%)			< 0.001^b^	
15–29	5 (1)	3 (< 1)		8 (1)
30–59	218 (62)	402 (49)		620 (53)
≥ 60	132(37)	417 (51)		549 (46)
Extent of disease,n(%)			0.278^b^	
Localized	58 (16)	154 (19)		212 (18)
Regional	254 (72)	558 (68)		812 (69)
Metastatic	28 (8)	85 (10)		113 (10)
Unknown	15 (4)	25 (3)		40 (3)
Area of residence, n(%)			< 0.001^b^	
Urban	134 (38)	437 (53)		571 (49)
Rural	98 (28)	327 (40)		425 (36)
Unknown	123 (35)	58 (7)		181 (15)

^a^ Wilcoxon Rank sum test

^b^ Chi-square test

**Table 2:** Characteristics of women targeted by the screening program (age 30–59) in urban and rural Chiang Mai who died of cervical cancer, 2003–2012

	Urban *N* = 159	Rural *N* = 222	*p*-value
**Characteristics**			
Median (IQR) age at diagnosis (year)	48 (45–53)	48 (44–52)	0.661^a^
Median (IQR) age at death (year)	51 (46–55)	50 (47–55)	0.905^a^
Extent of disease, n(%)			0.102^b^
Localized	28 (18)	54 (25)	
Regional/ Metastatic	127 (82)	160 (75)	

^a^ Wilcoxon Rank sum test

^b^ Chi-square test

### Cervical cancer mortality trends by age group

**Fig. 1:**
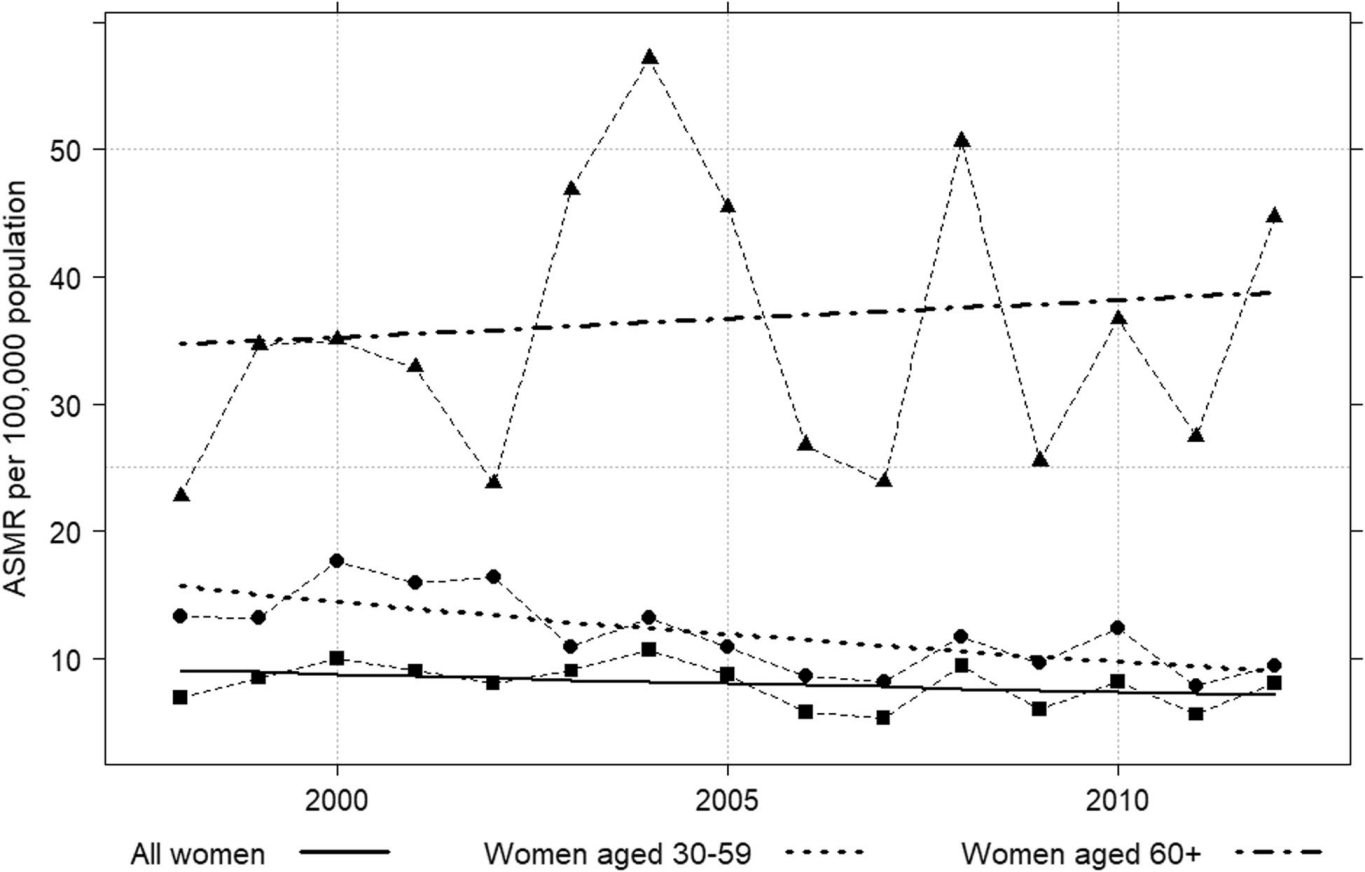
Trend in age-standardized mortality rate (ASMR) of cervical cancer in all women, screening target women and women aged 60 and older, 1998–2012

Table 3 shows the mortality rate of all women who died of cervical cancer. There was a significant decrease in the 5-year mortality from period I (1998–2002) to period III (2008–2012), with an APC of − 1.3 (95% CI: − 1.9 to − 0.7, *p* < 0.001). Fig. 1 shows the trend in cervical cancer mortality by age group from 1998 to 2012. The zero join point model was the best fit for describing the change in mortality of cervical cancer in all women. Based on the model, overall, cervical cancer mortality rates decreased from an ASMR of 9.1 per 100,000 women-years in 1998 to 7.1 per 100,000 women-years in 2012, an APC of − 1.7 (95% CI:-4.3 to 1.0). In women of screening target age, the mortality rates significantly decreased, from an ASMR of 15.6 per 100,000 in 1998 to 9.0 per 100,000 women-years in 2012, an APC of − 3.9 (95% CI: − 6.1, − 1.6). For women over 60, the rates remained stable, APC of 0.8 (95% CI: − 3.3 to 5.0) (Table 3). The mortality trend in women aged < 30 could not be described due to too few deaths in this age group from cervical cancer.

**Table 3:** Age standardized mortality rate (ASMR) and annual percent change (APC) of mortality rate

Category	ASMR	APC	95% CI	*p*-value
5-year period				
1998–2002	42.3	−1.3	−1.9 to −0.7	< 0.001
2003–2007	39.4			
2008–2012	37.1			
Age group	Year: 1998, 2012			
All age group	9.1, 7.1	−1.7	−4.3 to 1.0	0.2
30–59	15.6, 9.0	−3.9	−6.1 to −1.6	< 0.001
≥ 60	34.7, 38.8	0.8	−3.3 to 5.0	0.7
Area of residence	Year: 2003, 2012			
Urban				
30–59	10.1, 5.1	−7.6	−12.1 to −2.8	< 0.001
≥ 60	34.0, 21.6	−4.9	−11.5 to 2.1	0.1
Rural				
30–59	11.2, 15.6	3.7	−2.1 to 9.9	0.2
≥ 60	39.4, 53.1	3.4	−5.7 to 13.2	0.4

### Trends of cervical cancer mortality by area of residence

**Fig. 2:**
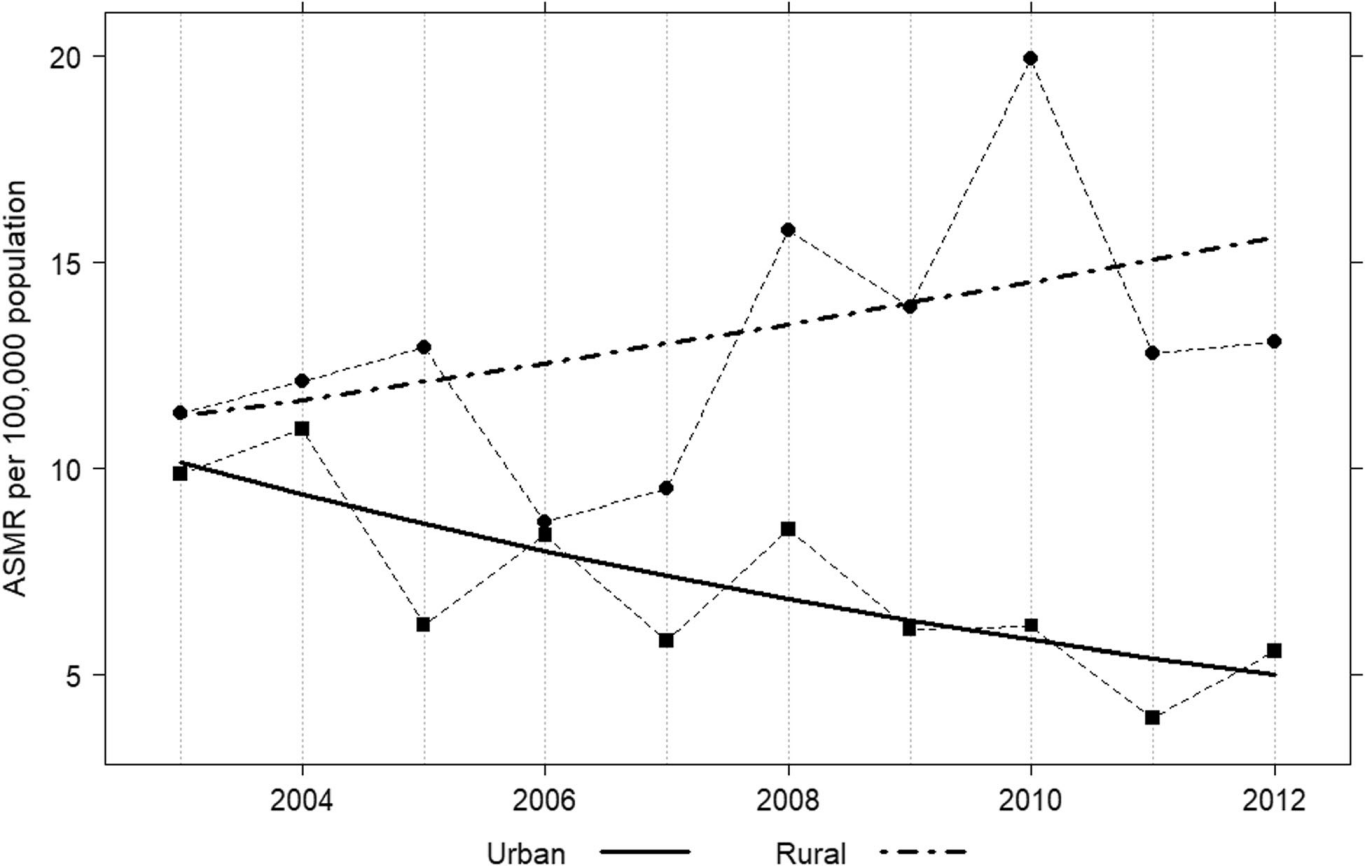
Trend in age-standardized mortality rate (ASMR) of cervical cancer in screening target women (aged 30–59 year) living in urban and rural areas, 2003–2012

More than 15 % had an unknown area in the period prior to the UHC since the area of residence data were not routinely recorded in this period. Therefore, the change of mortality by areas of residence were only be described in the era of UHC. Figure 2 shows the trends in cervical cancer mortality in screening target women (aged 30–59 years) during the period of UHC (2003–2012) by area of residence. A zero join point model was the best fit for the mortality rate of women of screening target age who lived in urban and rural areas. Based on the model, in urban residents, mortality rates from cervical cancer continuously decreased, from an ASMR of 10.1 per 100,000 women-years in 2003 to 5.1 per 100,000 women-years in 2012, an APC of − 7.6 (95% CI: − 12.1 to − 2.8, *p* < 0.001). This decrease was not seen among rural women, where the APC for the same period was − 4.9 (95% CI: − 11.5 to 2.1). Higher mortality rates were found in rural women of screening target age compared to their urban counterparts, at 11.2 per 100,000 women-years in 2003 and at 15.6 per 100,000 women-years in 2012 (Table 3).

## Discussion

Our study showed that cervical cancer mortality rates in all women of Chiang Mai decreased, from an ASMR of 9.1 per 100,000 women-years in 1998 to 7.1 per 100,000 women-years in 2012. These findings were consistent with previously published data showing that the mortality of cervical cancer of the upper northern Thai population decreased, from an ASMR of 7.3 per 100,000 women-years in 1998 to 4.6 per 100,000 women-years in 2012 [17]. These changes unfolded after UHC and screening programs for women aged 30–59 were introduced in Thailand and likely were a result of increasing access to these preventive services. In this analysis, we observed that reductions in cervical cancer mortality were particularly pronounced in women of screening target age group in Chiang Mai, Thailand, a change that was not seen in older populations. These findings are consistent with previously published studies showing a reduction in the incidence rate and increased survival in this Chiang Mai population [9, 10]. A study found that the proportion of metastatic cervical cancer remained large over three periods, period I: 1998–2002, period II: 2003–2007 and period 3: 2008–2012 in the women aged more than 60 [10]. This may explain the remaining high mortality rate in older population.

In some low-resource countries, a reduction in mortality rate of cervical cancer following the establishment of cervical cancer screening programs was not shown [18–20]. Whereas, a number of studies in high-income countries have illustrated an association between the implementation of screening programs and reductions in cervical cancer mortality [21, 22]. A recent study [22] reported that cervical cancer screening every 3 years in women aged 25–49 years and every 5 years in women aged 50–64 resulted in a reduction in the mortality rate of cervical cancer of 70% (95% CI: 66–73%) across women of all age groups.

Although, in our study, the results showed the reduction of cervical cancer mortality in women of screening target age, this was not equitably distributed, and a disproportionate decrease in cervical cancer mortality rates in urban residents drove overall reductions in cervical cancer mortality, whereas rates in rural women remained static over the same period.

Thus, this stark difference in the change of mortality rate was likely a result of disparities in coverage of screening and access to health care. In other low-resource countries, there is evidence that increasing coverage of cervical cancer screening for women correlated with a reduction of mortality from cervical cancer in Mexico [23]. In Thailand, a number of surveys have indicated that coverage of cervical cancer screening was between 38 and 63% between 2003 and 2006 [24, 25] and showed that non-municipal areas, defined as rural areas were more likely to have higher proportion of women who ever had at least once-in-a-lifetime cervical cancer screening compared to those who lived in municipal (urban areas) [26]. However, the compliance to the screening guideline by areas of residence has not been reported in this study. The low compliance may be a reason behind unchanged mortality rate in rural areas. There was a study in urban Northeastern Thailand that showed approximately 65.4% of the women were considered to be compliance to cervical cancer screening [27] but there was no study that reported this information for rural area.

The cervical cancer mortality by areas of residence may be in concordance with its incidence. The supplementary Figure 1 shows the reduction in incidence of cervical cancer in urban but stable and higher in rural in the same population during the same period with this present study. For example, the decline in mortality up to 2006 and the rise in mortality rate after 2006 were align with the change of incidence.

The urban-rural disparities were also shown in other health outcomes in Thailand. A study showed difference pattern of diseases in lower incomes area compared to higher incomes areas for instance malaria, goiter, kidney stone, and tuberculosis occurred more with lower incomes, whereas allergic conditions and migraine were found more with higher incomes. Inequality in these health outcomes were found to be associated with older age, low education, and residence in the rural Northeast and rural North of Thailand [28]. Our study demonstrated that cervical cancer mortality is one further manifestation of these urban-rural disparities.

The reasons behind this disparity in change of mortality remain unclear, one that likely is a result of disparities in access to health care included access to screening and compliance to the screening guideline. Another potential reason is lack of knowledge of cervical cancer prevention. A positive attitude towards cervical cancer screening was associated knowledge about cervical cancer screening [29, 30]. Moreover, the knowledge level about the risks, the disease and the screening for cervical cancer was significantly associated with cervical cancer screening compliance [27]. The proportion of a poor attitude regarding cervical cancer screening remained large in rural community women (22.6%) [31].

One sustainable way to improve coverage of health care access and cervical cancer screening in this population is to educate people in particular those who live in rural area about the benefit of cervical cancer screening. Moreover, a potential communication model for unique population context needs to be formed for promoting and increasing the access of UHC and cervical cancer screening. The communication through the health volunteer in the community which are prevalent in Thailand could be a good option.

The accuracy of screening approach is one of the factors to achieve the goal of reduction in mortality rate from cervical cancer. Sankaranarayanan et al. [32] found that a single round of HPV testing was associated with a significant reduction in cases of advanced cervical cancers and deaths from cervical cancer (HR = 0.52 (95% CI, 0.33 to 0.83). One analysis done in Thailand suggests that primary HPV testing every 5 years should be considered as an optimal strategy to detect early stage of cervical cancer because of more cost-effective than cytology testing every 5 years for cervical cancer [33]. In 2020, this approach has been launched in some areas of Thailand for cervical cancer screening, with scale-up occurring in 2021, with planned inclusion of the entire country in 2022 [34]. This screening method will likely further improve the accuracy of detection of cervical cancer at the early stages [35]. The new guidelines for HPV screening to prevent cervical cancer are expected to bring wider coverage in Thailand and further changes. Yet these improvements in screening will likely continue to be inequitably distributed, primarily benefitting urban residents, and further evaluations for these inequities are needed.

This study had several limitations. One is potential inaccuracy of cause-of-death data registered in the vital registration (VR) system of Thailand, which is the most important source of mortality data for the whole country [36]. However, the Thai Ministry of Public Health (MOPH) is aware of this issue and has used the Verbal Autopsy (VA) method since 2005 to improve the quality of reporting the cause of death [37]. Moreover, the cause of death among deaths occurring in hospitals of patients used in our dataset were also verified using data from the Chiang Mai Cancer Registry.

The definition of urban versus rural residency is another limitation of our study. We could not directly compare the results by area of residence with the previous studies that classified urban areas on the basis of the Official Statistics Registration Systems, with urban areas being municipal areas and rural areas as non-municipal ones [3, 26]. Our study could not use this definition because the available data on area of residence from the Cancer Registry were recorded in difference form.

Another limitation is that the results of our study was based on only population-level data from the Chiang Mai Province in Northern Thailand, which represents only about 15% of the entire population of Northern Thailand. Thus, our findings may not be generalizable to all northern Thai women, particularly given the ethnic diversity of the area compared to the rest of the country. However, our analysis likely is an accurate representation of the situation in Chiang Mai province as it relies on all reported cervical cancer deaths here, minimizing selection bias. The data was collected by a cancer registry with the active method since the establishment of the cancer registry in 1986. Therefore, the data is generally considered consistent and complete, with more than 95% of diagnoses confirmed by histologic verification (HV) and less than 1% on the sole basis of a death certificate.

This is the first study examining the changes in cervical cancer mortality by area of residence in Thailand, showing that the mortality rate remained high in rural areas, with the overall reduction in cervical cancer mortality in Chiang Mai being driven largely by a reduction in death rates in urban women.

## Conclusion

The mortality rate of cervical cancer decreased in women of screening target age in Chiang Mai, Thailand, following the introduction of UHC. The reductions primarily occurred in urban areas but remained unchanged in rural areas. Our findings underscore the need to further examine the reasons underpinning this disparity, one that likely is a result in inequitable access to either screening program or health care service between urban and rural Chiang Mai. The knowledge about cervical cancer screening may be a key to improve attitude toward cervical cancer screening in rural Chiang Mai. These results suggest that without further evaluation of and addressing these disparities, the beneficiaries from current and potentially future cervical cancer prevention programs will primarily be urban Thai women.

## Supplementary Information

**Additional file 1: Supplementary Figure 1.** Trend in age-standardized incidence rate (ASIR) of cervical cancer in screening target women (aged 30–59 year) living in urban and rural areas, 1998–2012.

## Data Availability

The datasets used and/or analyzed during the current study are available from the corresponding author on reasonable request.

## Abbreviations

UHC: Universal Health Coverage
NHSO: National Health Security Office
Pap Smear: Papanicolaou smear
VIA: Visual inspection with acetic acid
ICD-10: International Classification of Diseases 10th edition
ASR: Age-standardized rates
ASMR: Age-standardized mortality rate
ASIR: Age-standardized incidence rate
APC: Annual percent change
VR: Vital registration
MOPH: Ministry of Public Health
VA: Verbal Autopsy
HV: Histology verification
DCO: Death certificate only case
