# Supplementary figures and images for: Disparities in the change of cervical cancer mortality rate between urban and rural Chiang Mai in the era of universal health care and the Thai national screening program
Source: Int J Equity Health. 2021 Jul 29;20:175. doi: 10.1186/s12939-021-01515-1 (PMC8323343; doi:10.1186/s12939-021-01515-1)

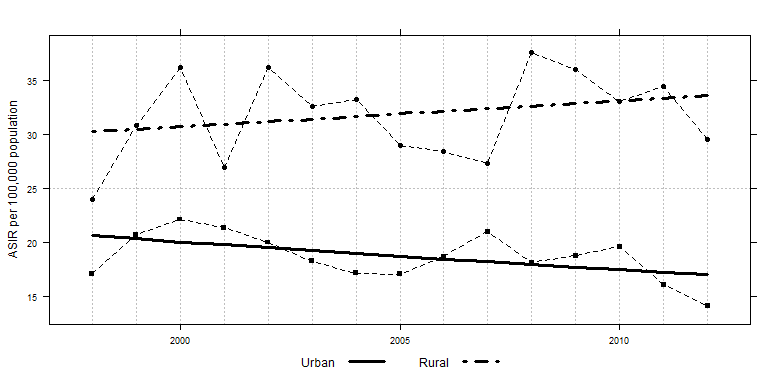

Supplement: Supplementary file 1 — Additional file 1: Supplementary Figure 1. Trend in age-standardized incidence rate (ASIR) of cervical cancer in screening target women (aged 30–59 year) living in urban and rural areas, 1998–2012. [file 12939_2021_1515_MOESM1_ESM.tif]
